# Identification of Tregs-Related Genes with Molecular Patterns in Patients with Systemic Sclerosis Related to ILD

**DOI:** 10.3390/biom13030535

**Published:** 2023-03-15

**Authors:** Jiao Luo, Dongdong Li, Lili Jiang, Chunhua Shi, Lihua Duan

**Affiliations:** 1 Department of Rheumatology and Clinical Immunology, Jiangxi Provincial People’s Hospital, The First Affiliated Hospital of Nanchang Medical College, Nanchang 330000, China; 2 Medical College of Nanchang University, Nanchang 330000, China

**Keywords:** Treg cells, SSc, genes

## Abstract

Background: Systemic Sclerosis (SSc) is an autoimmune disease that is characterized by vasculopathy, digital ulcers, Raynaud’s phenomenon, renal failure, pulmonary arterial hypertension, and fibrosis. Regulatory T (Treg) cell subsets have recently been found to play crucial roles in SSc with interstitial lung disease (ILD) pathogenesis. This study investigates the molecular mechanism of Treg-related genes in SSc patients through bioinformatic analyses. Methods: The GSE181228 dataset of SSc was used in this study. CIBERSORT was used for assessing the category and proportions of immune cells in SSc. Random forest and least absolute shrinkage and selection operator (LASSO) regression analysis were used to select the hub Treg-related genes. Results: Through bioinformatic analyses, LIPN and CLEC4D were selected as hub Treg-regulated genes. The diagnostic power of the two genes separately for SSc was 0.824 and 0.826. LIPN was associated with the pathway of aminoacyl−tRNA biosynthesis, Primary immunodeficiency, DNA replication, etc. The expression of CLEC4D was associated with the pathway of Neutrophil extracellular trap formation, PPAR signaling pathway, Staphylococcus aureus infection, Systemic lupus erythematosus, TNF signaling pathway, and Toll−like receptor signaling pathway. Conclusion: Through bioinformatic analyses, we identified two Treg-related hub genes (LIPN, CLEC4D) that are mainly involved in the immune response and metabolism of Tregs in SSc with ILD. Moreover, our findings may provide the potential for studying the molecular mechanism of SSc with ILD.

## 1. Introduction

Systemic Sclerosis (SSc) as an autoimmune disease has severe clinical manifestations and a high mortality rate, and treatment for this disease is minimal and ineffective [1]. SSc is characterized by vasculopathy, digital ulcers, Raynaud’s phenomenon, renal failure, pulmonary arterial hypertension, and fibrosis [2,3]. Fibrosis, as a hallmark of SSc, frequently involves the lung, manifesting as interstitial lung disease (ILD) [4,5]. It is the most serious complication associated with SSc and is the top cause of death associated with SSc [6]. Clinical and pathologic manifestations of SSc are due to abnormalities in the innate and adaptive immune systems, which result in the production of autoantibodies and cell-mediated autoimmunity. Then, the accumulation of collagen and other matrix components in the skin and internal organs occurs due to microvascular epitheliopathy and fibroblast dysfunction [7]. In SSc with ILD, fibrosis may result from an interplay between autoimmunity, inflammation, and epithelial and vascular injury [8]. However, the pathogenesis of fibrosis often lacks insight into the interactions between key players. Regulatory T (Treg) cell subsets have recently been found to play crucial roles in SSc pathogenesis [9,10,11]. The importance of Tregs for maintaining immune homeostasis and self-tolerance is increasingly recognized. Most studies reported that SSc patients had reduced frequency and/or impairment of circulating Tregs [12]. Fenoglio et al. found an imbalance between circulating Th17 cells and Treg cells (Tregs) in patients with SSc, with an increased proportion of Th17 cells and a decrease in both CD4^+^CD25^+^CD127^−^ and CD8^+^CD28^−^Treg cells [13]. Also, patients with high computed tomography scores for ILD had elevated Treg cells [14,15]. Nevertheless, studies of Tregs’ phenotype and function are rare. Little is known about the regulatory mechanisms of Tregs alteration in SSc with ILD. Identifying biomarkers related to Tregs will facilitate the exploration of immune infiltration mechanisms of SSc with ILD.

Bioinformatics-based studies of the contribution of genes related to Tregs of SSc with ILD have not been conducted yet. To explore the effect of Treg cells and identify potential biomarkers of SSc with ILD, WGCNA was performed using gene expression data in the peripheral blood of SSc with ILD. The T-cell compositions of samples were calculated using the CIBERSORT algorithm. We then identified Treg-related genes from important modules and genes related to Tregs infiltration levels, and machine learning was used to identify hub Treg-related genes. Identifying Treg-related genes of SSc with ILD may provide potential pathogenesis and therapeutic targets of SSc with ILD.

## 2. Materials and Methods

### 2.1. Expression Data Download and Processing

The dataset GSE181228 was downloaded from the GEO (https://www.ncbi.nlm.nih.gov/geo/ accessed on 5 October 2022) database. The GSE181228 dataset was last updated on 1 February 2022 and annotated on platform GPL24676. Samples were obtained from peripheral blood and contained 134 SSc with ILD patients (untreated) and 45 healthy controls. Expression data of GSE181228 were DESeq2 normalized and log2 transformed by the uploader. Genes that corresponded to multiple probes were averaged after annotation. Genes with the expression of 0 in more than 30 samples were excluded. Since the data of this study were obtained from public databases, ethics committee approval was not required.

### 2.2. Differentially Expressed Genes (DEGs)

DEGs between the SSc with ILD patients and healthy groups were analyzed using the R package “limma” [16]. Genes with adj. *p* < 0.05 and an absolute value of log2 (fold change(FC)) > 1 were identified as DEGs. The R package “heatmap” and “ggplot2” were used to map volcanoes and heatmaps.

### 2.3. Immune Infiltration Analysis for the Datasets and Weighted Gene Co-Expression Network Analysis (WGCNA)

In this study, the relative expression of 22 immune cells in each sample was determined using the “CIBERSORT” (R package) [17]. Cells with an expression of 0, which were more than 50% in the sample, were excluded. Heat maps and box plots were plotted using the R package “heatmap” and “ggplot2”. Weighted correlation network analysis was performed using the R package “WGCNA” [18]. The samples were clustered, and outlier samples were excluded. To exclude highly correlated genes that did not vary significantly, the MAD method was used to select the 5000 genes with the largest absolute median difference. The correlation matrix was constructed, and a weighted adjacency matrix was generated. Suitable β values were selected to obtain the topological overlap matrix (TOM). Modules of a minimum of 30 genes were constructed using average linkage hierarchical clustering and module dendrograms. To measure the correlation between genes and immune cells, gene significance (GS) was calculated to determine the significance of each module. A threshold of more than 0.25 was used to merge similar modules [19]. The most correlated modules with Tregs were identified, and the intersection between Tregs’ most correlated module genes and DEGs was identified as Tregs-related DEGs (TDEGs).

### 2.4. Identification of Hub Genes

Based on differentially expressed feature genes, least absolute shrinkage and selection operator (LASSO) regression analysis [20] was performed using the R package “glmnet” [21], and the variables corresponding to the value of the penalty parameter lambda.1se were selected as marker genes using 10-fold cross-validation. The R package “randomForest” was used to screen important key genes in a random forest (RF) classifier with several binary trees initially ranging from 1 to 100 cycles, respectively. Binary trees were selected based on the lowest value of the error rate, and decision trees were selected based on model stability, thus constructing the random forest model. The random forest uses 10-fold cross-validation repeated 5 times to select the optimal number of marker genes. Intersect genes of two algorithms were selected as hub genes. Hub genes were used to construct logistic regression models using the R package “glmnet”. ROC curve analysis was performed using the R package pROC to calculate the area under the curve (AUC) and assess the diagnostic ability of the model [22].

### 2.5. Gene Set Enrichment Analysis (GSEA) of Hub Genes

Samples were divided into two groups based on the median expression of the signature genes, and GSEA analysis [23] was performed using the “gseKEGG” in the R package “clusterProfiler”. A *p*-value < 0.05 was indicative of statistical significance. The number of permutations was set to 1000, and the permutation type was set as “gene list”. The most significantly enriched pathway was selected based on the enrichment score.

### 2.6. Statistical Analysis

R software (version 4.1.3) was used to perform statistical analysis. Student’s *t*-test (two-tailed) was used to determine the statistical significance for both groups. A logistic regression algorithm was used to build a prediction model. Roc curve analysis and the area under the curve were calculated. A *p* < 0.05 was considered a statistically significant difference.

## 3. Results

### 3.1. Identification of Differentially Expressed Genes

**Figure 1:**
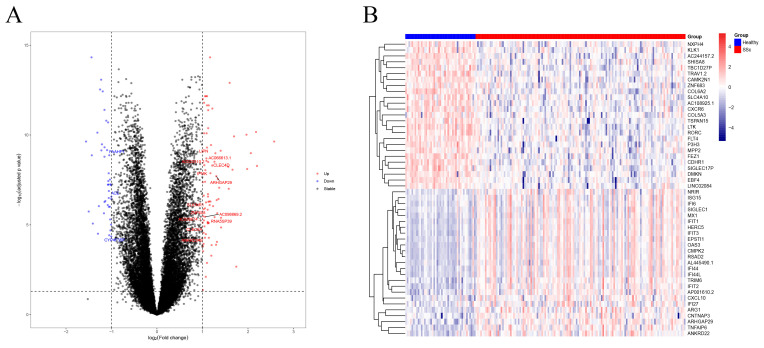
Identification of DEGs. (**A**) Volcano plot for differentially expressed genes between normal and patients with SSc-related ILD. (**B**) The heatmap of the top 25 up-regulated and 25 down-regulated DEGs.

An expression matrix containing 179 samples was obtained from the GSE150910. Differential expression analysis was performed between 134 SSc with ILD patient groups and 45 healthy groups, and a total of 125 differentially expressed genes (DEGs) were obtained, 83 up-regulated genes and 42 down-regulated genes. One hundred twenty-five DEGs were visualized by volcano map (Figure 1A). The differentially expressed genes with log2 ratio and adjusted *p*-value are shown in Table S1, and the most significantly differentially up- and down-regulated 25 DEGs each were visualized by heatmap (Figure 1B).

### 3.2. Immune Cell Landscape

**Figure 2:**
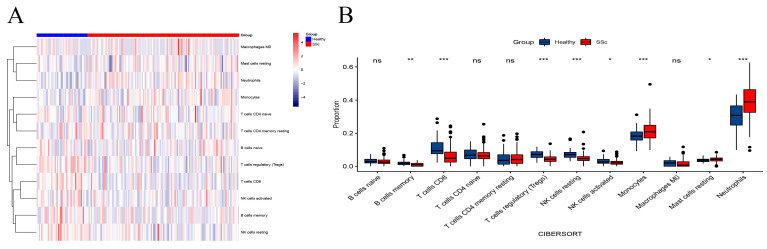
Immune landscape analysis. (**A**) The heatmap of 12 immune cells of healthy cohorts and SSc with ILD in GSE181228. (**B**) The different expressions between each of the immune cells. * *p* < 0.05 ** *p* < 0.01 *** *p* < 0.001.

A total of 12 cell types were obtained, and the expression of 12 immune cell types between the SSc with ILD patient group and the healthy group was visualized by heatmap (Figure 2A). Compared to the healthy group, the expression of peripheral cells (B cells memory, CD8^+^ T cells, T cells regulatory (Tregs), NK cells resting, NK cells activated) was significantly higher in the SSc with ILD patient group. On the contrary, the expression of infiltration cells (Monocytes, Mast cells resting, Neutrophils) of SSc with ILD patients was significantly decreased compared to the healthy group (Figure 2B).

### 3.3. The WGCNA Co-Expression Network and Identify Differential Tregs-Related DEGs (TDEGs)

**Figure 3:**
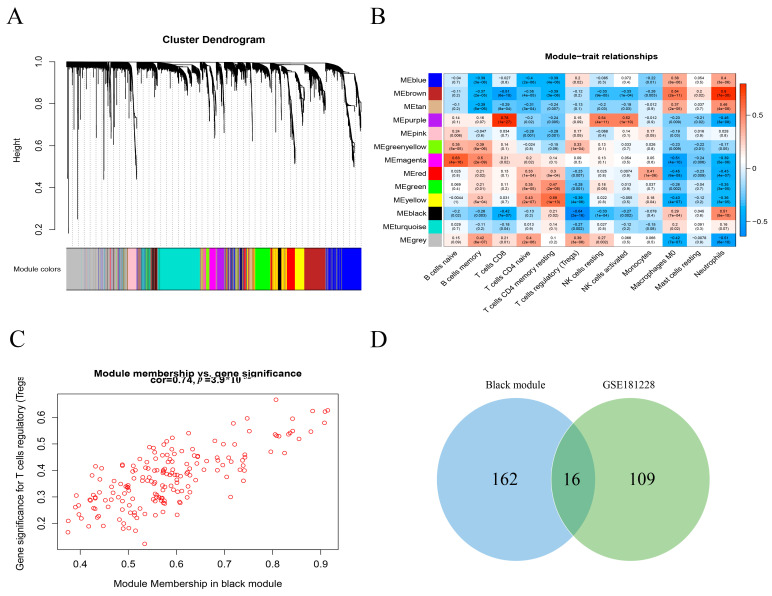
Identification of Treg-related genes. (**A**) Genes were grouped into various modules by hierarchical clustering according to dynamic tree cut and merged when the module’s correlation > 0.25. Different colors represent different modules. (**B**) Correlation between modules and immune cells. (**C**) A scatter plot of genes in the black module were Treg-related. Each green dot represents a gene. (**D**) 16 Treg-regulated genes were visualized by a Venn diagram, shown as the overlap between DEGs and genes in the black module by weighted correlation network analysis (WGCNA).

Using sample clustering, three outlier samples were first excluded from this study. A scale-free network was constructed by selecting β = 6 (no scale R2 = 0.943) as the soft threshold. A total of 5000 genes were grouped into 12 modules (Figure 3A). The black module mostly correlated with the Tregs (Figure 3B). The significance of genes in the black module for Tregs is shown in Figure 3C (0.74, *p* < 0.001). TDEGs were obtained by taking the intersection of Tregs-related genes and DEGs (Figure 3D).

### 3.4. Identify Hub Genes

**Figure 4:**
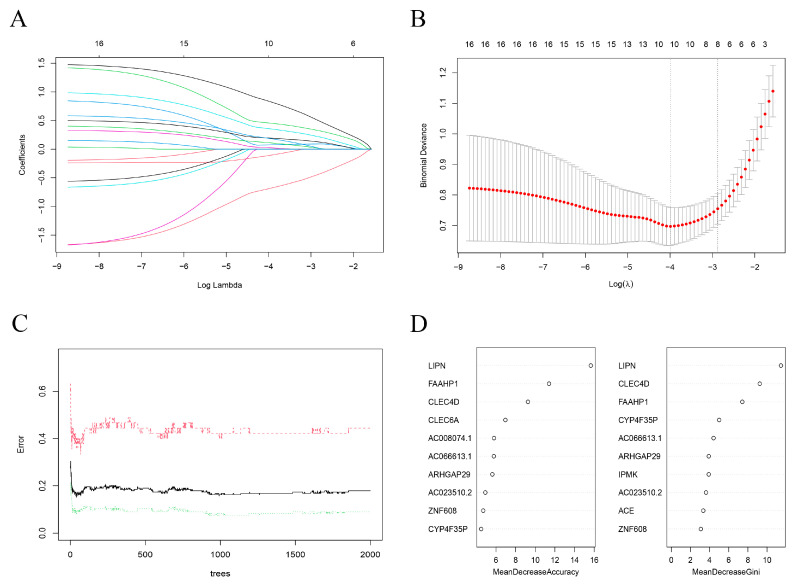
Two machine learning algorithms to screen Treg-related genes. (**A**) Lasso coefficient curves for TDEGs. (**B**) Ten-fold cross-validation of optimal parameter selection in lasso regression analysis. (**C**) The relationship between the number of decision trees and the model error for the RF algorithm for screening hub genes. The *X*-axis represents the number of decision trees. The *Y*-axis represents the error rate of the constructed model. (**D**). Mean decrease accuracy and mean decrease Gini coefficients correspond to the ten characteristic genes obtained from the RF algorithm. The *X*-axis represents the coefficients, and the *Y*-axis represents the genes.

**Figure 5:**
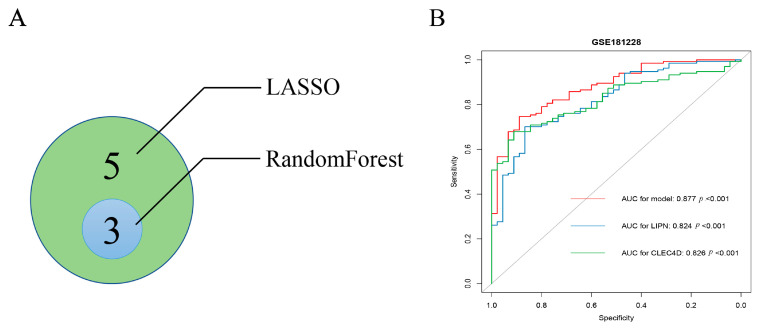
Identification of hub Treg-related genes. (**A**) Three hub Treg-related genes were visualized by a Venn diagram, shown as the overlap of genes selected from two machine learning algorithms. (**B**) ROC curve of hub Treg-related genes including LIPN, CLEC4D, and model.

Eight genes were identified by LASSO regression analysis (the optimal lambda.1se was 0.056) in 16 TDEGs (Figure 4A,B). Sixteen TDEGs were most stable with a binomial tree of 7 and a decision tree of 400 using the random forest algorithm (Figure 4C). The top 10 significant genes were ranked according to accuracy and Gini coefficient (Figure 4D). A 10-fold cross-validation using random forest was repeated 5 times, and 3 genes were finally selected. The intersection of the two algorithms was taken to obtain a total of three significant hub genes (LIPN, CLEC4D, FAAHP1) (Figure 5A). According to GeneCards (https://www.genecards.org/ accessed on 5 October 2022) database, FAAHP1 is a pseudogene, so this gene was eliminated in this study. A prediction model was developed using a logistic regression algorithm using two genes (LIPN, CLEC4D) with the equation (y = −15.598 + 1.512exp + 1.065exp). The diagnostic power of the two genes separately for SSc with ILD was displayed in Figure 5B (LIPN, CLEC4D; 0.824, 0.826). The diagnostic power of the model for SSc with ILD was 0.877 (Figure 5B).

### 3.5. Signaling Pathways Associated with Hub Genes by GSEA

**Figure 6:**
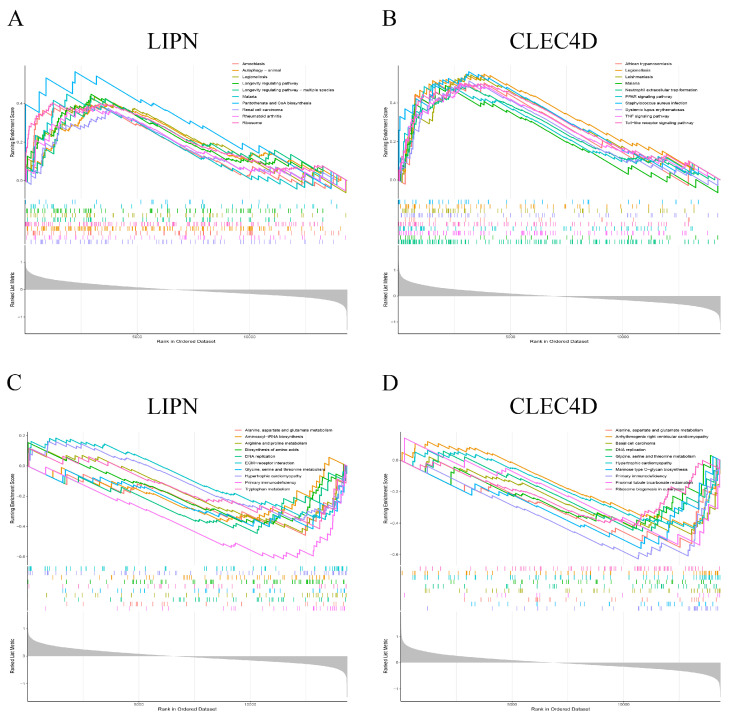
GSEA of hub Treg-related genes. (**A**) Ten upregulated pathways of LIPN. (**B**) Ten upregulated pathways of CLEC4D. (**C**) Ten downregulated pathways of LIPN. (**D**) Ten downregulated pathways of CLEC4D.

In this study, the functions of hub genes were explored using GSEA. Ten significantly up- and down-regulated enrichment KEGG pathways of hub genes were selected based on enrichment scores. The KEGG pathways of hub genes are shown in Tables S2 and S3. The expression of LIPN was associated with the up-regulated pathway of amoebiasis, Autophagy-animal, Legionellosis, Longevity regulating pathway, Longevity regulating pathway-multiple species, Malaria, Pantothenate and CoA biosynthesis, Renal cell carcinoma, Rheumatoid arthritis, and Ribosome. In contrast, the expression of LIPN was down-regulated in pathways of alanine, aspartate, and glutamate metabolism, aminoacyl-tRNA biosynthesis, arginine and proline metabolism, biosynthesis of amino acids, DNA replication, ECM-receptor interaction, glycine, serine and threonine metabolism, Hypertrophic cardiomyopathy, Primary immunodeficiency, and Tryptophan metabolism (Figure 6C). The expression of CLEC4D was associated with the up-regulated pathway of African trypanosomiasis, Legionellosis, Leishmaniasis, Malaria, Neutrophil extracellular trap formation, PPAR signaling pathway, Staphylococcus aureus infection, Systemic lupus erythematosus, TNF signaling pathway, and Toll-like receptor signaling pathway. The expression of CLEC4D was associated with the down-regulated pathway of alanine, aspartate, and glutamate metabolism, Arrhythmogenic right ventricular cardiomyopathy, Basal cell carcinoma, DNA replication, glycine, serine and threonine metabolism, Hypertrophic cardiomyopathy, Mannose type O-glycan biosynthesis, Proximal tubule bicarbonate reclamation, Proximal tubule bicarbonate reclamation, and Ribosome biogenesis in eukaryotes (Figure 6D).

## 4. Discussion

Systemic sclerosis (SSc) is an autoimmune disease with high mortality. Pulmonary fibrosis is the most common and serious clinical manifestation of the disease and is also the main cause of death. The pathophysiology of SSc with ILD is complex and unclear. Due to immune dysfunctions, SSc is characterized by autoimmunity, vasculopathy, and fibrosis. The complex interaction and activation of immune cells are key factors in the formation of fibrosis. Tregs, as a type of CD4 + T cells, have been demonstrated to play an important role in SSc with different mechanisms. In the mouse model of bleomycin-induced pulmonary fibrosis, CD4 + CD25highFoxP3+ cells in the lung were increased after IL-2 complex treatment associated with aggravated lung fibrosis [24]. The role of Tregs in the SSc-ILD is more and more recognized. However, the regulatory mechanisms of T cells in SSc remain poorly understood.

In this study, we used bioinformatics methods to explore the role of Tregs in the SSc with ILD and identify the hub gene of Tregs in SSc with ILD and effective diagnostic biomarkers for SSc with ILD. DEGs were obtained from peripheral blood and contained 134 SSc with ILD patients (untreated) and 45 healthy controls. Compared to the healthy group, the expression of peripheral cells (B cells memory, CD8^+^ T cells, T cells regulatory (Tregs), NK cells resting, NK cells activated) was significantly higher in the SSc with ILD patient group. On the contrary, the expression of infiltration cells (Monocytes, Mast cells resting, Neutrophils) of SSc with ILD patients was significantly decreased compared to the healthy group. Some reports showed that Treg cells without immunosuppressive functions have increased in number, while those with immunosuppressive functions have decreased among Treg cells of SSc patients [10]. SSc patients with active disease exhibit upregulation of FOXP3 gene expression in Treg cells [25]. In this study, Tregs also were significantly higher in the SSc with ILD patients. WGCNA was used to extract Tregs-related genes. Then, TDEGs were obtained by taking the intersection of Tregs-related genes and DEGs. A commonly used algorithm, LASSO analysis which is a machine learning–based algorithm, has been demonstrated to yield clinical efficacy [26,27]. For RF analysis, there is no restriction on variable conditions, which makes it an appropriate ensemble learning algorithm and machine learning method [28]. The RF method can be used to predict continuous variables with no obvious deviations from the prediction [29]. We used both classical algorithms to select hub genes of Tregs in SSc with ILD. Finally, LIPN and CLEC4D were obtained from the intersection of the LASSO regression analysis and random forest based on TDEGs. The LIPN and CLDE4D were demonstrated to be hub genes of Tregs for SSc with ILD. The KEGG pathway of LIPN and CLEC4D was analyzed by GSEA. The expression of LIPN was associated with an up-regulated pathway of amoebiasis, Autophagy-animal, Rheumatoid arthritis, and so on. The expression of LIPN was associated with a down-regulated pathway of aminoacyl-tRNA biosynthesis, Primary immunodeficiency, DNA replication, and so on. The expression of CLEC4D was associated with an up-regulated pathway of Neutrophil extracellular trap formation, PPAR signaling pathway, Staphylococcus aureus infection, Systemic lupus erythematosus, TNF signaling pathway, and Toll−like receptor signaling pathway. The expression of CLEC4D was associated with a down-regulated pathway of alanine, aspartate, and glutamate metabolism, Arrhythmogenic right ventricular cardiomyopathy, Basal cell carcinoma, DNA replication, and so on. Known as LIPN (Rv2970c), it belongs to the Lip family of M. tuberculosis H37Rv and is homologous to the human hormone-sensitive lipase. In addition to its preference for short carbon chain substrates [30], Shirli et al. reported that the LIPN gene encoding epidermal lipase N has been linked to congenital ichthyosis with a late-onset form associated with autosomal-recessive inheritance [31]. In another paper, gestational diabetes mellitus was associated with lipolysis-related genes such as LIPN [32]. However, there are few studies on LIPN in autoimmune diseases, especially SSc with ILD. In this study, LIPN was demonstrated to be correlated with amino acid metabolism. LIPN may be a hub gene to affect the metabolism of Tregs. CLDE4D, as a C-type (Ca^2+^-dependent) lectin (CLEC) receptor (CLEC), has potential regulatory effects on immune cell trafficking, which is essential in innate pattern recognition [33]. CLEC4D, as a key component of anti-mycobacterial immunity, was expressed by myeloid cells [34]. It has been demonstrated that the deficiency of CLEC4D in the gut promotes the development of colitis by impairing antifungal immune responses [35]. The function of CLEC4D in SSc with ILD, especially in Tregs, was not studied. In this research, CLEC4D was a Tregs related-gene and associated with the immune-related pathway. It means that CLEC4D was associated with the immune function of Tregs. Meanwhile, there was good diagnostic power of LIPN and CLEC4D for SSc with ILD. CLEC4D and LIPN may play a key role in SSc with ILD by affecting the function of Tregs. There are still limitations in this study that we cannot ignore. Generally, the ratio of women to men in SSc ranges from 3:1 to 7:1 [36]. In this study, the female SSc patients were up to 73.1%. As we could not get clinical information on the samples from the public database, gender might be a confounding variable in the analyses to impact the results of the differential analysis. In addition, this study was performed only between SSc with ILD and healthy controls. Patients with SSc were not included in the study, so the results could not be verified in SSc and SSc with ILD. The study should have included more forms of SSc patients. Nevertheless, these two genes have not been validated in other gene sets. LIPN and CLEC4D need further examination in in vivo or in vitro experiments; however, this gives us a direction. The mechanism of LIPN and CLEC4D for SSc needs to be further explored.

In conclusion, this study first analyzed and assessed molecular patterns of Tregs-related genes of SSc with ILD using bioinformatics methods. Our findings explored two Tregs-related genes of SSc (LIPN and CLEC4D). The Treg-related hub genes were mainly involved in amino acid metabolism and inflammatory pathways of Tregs in SSc with ILD. In addition, the analysis of the sensitivity and specificity of two Tregs-related hub genes unveiled that they may be potential biomarkers for SSc with ILD.

## Data Availability

The dataset GSE181228 was downloaded from the GEO (https://www.ncbi.nlm.nih.gov/geo/) (accessed on 5 Ocotber 2022).

## Supplementary Materials

The following supporting information can be downloaded at: https://www.mdpi.com/article/10.3390/biom13030535/s1, Table S1: The differentially expressed genes; Table S2: The KEGG pathways of LIPN; Table S3: The KEGG pathways of CLEC4D.
